# DNA methylation in Friedreich ataxia silences expression of frataxin isoform E

**DOI:** 10.1038/s41598-022-09002-5

**Published:** 2022-03-23

**Authors:** Layne N. Rodden, Kaitlyn M. Gilliam, Christina Lam, Teerapat Rojsajjakul, Clementina Mesaros, Chiara Dionisi, Mark Pook, Massimo Pandolfo, David R. Lynch, Ian A. Blair, Sanjay I. Bidichandani

**Affiliations:** 1 Department of Pediatrics, University of Oklahoma Health Sciences Center, OU Children’s Physician Building, Suite 12100, 1200 Children’s Avenue, Oklahoma City, OK 73104 USA; 2 Oklahoma Center for Neuroscience, University of Oklahoma Health Sciences Center, Oklahoma City, OK USA; 3 Department of Systems Pharmacology and Translational Therapeutics, Perelman School of Medicine, University of Pennsylvania, Philadelphia, PA USA; 4 Université Libre de Bruxelles (ULB), Brussels, Belgium; 5 Division of Biosciences, Department of Life Sciences, College of Health and Life Sciences, Brunel University London, Uxbridge, UK; 6 Department of Neurology and Neurosurgery, McGill University, Montreal, QC Canada; 7 Division of Neurology, The Children’s Hospital of Philadelphia, Philadelphia, PA USA; 8 Department of Biochemistry and Molecular Biology, University of Oklahoma Health Sciences Center, Oklahoma City, OK USA

**Keywords:** Genetics, Neurology

## Abstract

Epigenetic silencing in Friedreich ataxia (FRDA), induced by an expanded GAA triplet-repeat in intron 1 of the *FXN* gene, results in deficiency of the mitochondrial protein, frataxin. A lesser known extramitochondrial isoform of frataxin detected in erythrocytes, frataxin-E, is encoded via an alternate transcript (*FXN-E*) originating in intron 1 that lacks a mitochondrial targeting sequence. We show that *FXN-E* is deficient in FRDA, including in patient-derived cell lines, iPS-derived proprioceptive neurons, and tissues from a humanized mouse model. In a series of FRDA patients, deficiency of frataxin-E protein correlated with the length of the expanded GAA triplet-repeat, and with repeat-induced DNA hypermethylation that occurs in close proximity to the intronic origin of *FXN-E*. CRISPR-induced epimodification to mimic DNA hypermethylation seen in FRDA reproduced *FXN-E* transcriptional deficiency. Deficiency of frataxin E is a consequence of FRDA-specific epigenetic silencing, and therapeutic strategies may need to address this deficiency.

## Introduction

Friedreich ataxia (FRDA) is an autosomal recessive condition, characterized by progressive ataxia, dysarthria, weakness, fatigue and cardiomyopathy^1^. There is currently no effective disease-modifying therapy and the relentless progression results in considerable morbidity and premature mortality in the third or fourth decade of life^2–5^. The vast majority of patients inherit an expanded GAA triplet-repeat sequence in intron 1 of the *FXN* gene from each parent that ranges in size from 100 to 1500 triplets (versus < 30 in non-FRDA alleles)^3,6–9^. Expanded *FXN* genes develop repeat-dependent epigenetic silencing signals, including repressive histone marks and DNA hypermethylation, which are predominantly localized in intron 1^10–24^. This leads to repeat-dependent transcriptional deficiency of the canonical *FXN* transcript that encodes an essential mitochondrial protein, frataxin^20,25–27^. Initiation of this transcript occurs upstream of coding exon 1, which encodes most of the mitochondrial targeting sequence^6^. Epigenetic silencing and ensuing transcriptional deficiency of this *FXN* transcript occur via a combination of deficient transcriptional initiation and elongation, characterized by depletion of RNA polymerase at the transcription start site located upstream of exon 1, its stalling in exon 1, and reduced progression through intron 1^16–18,20,28,29^. However, the human *FXN* locus also codes for a less characterized, non-mitochondrial version of frataxin (frataxin-E) that is expressed in erythrocytes, which lack mitochondria^30^. It is unclear if this non-canonical product of the *FXN* gene is expressed in cell types that are relevant to FRDA pathogenesis, and how the epigenetic silencing signals in FRDA affect its expression. We show that the transcript predicted to encode frataxin-E (*FXN-E*) originates in intron 1 of the *FXN* gene in close proximity to the site of repeat-induced DNA hypermethylation in FRDA. *FXN-E* is deficient in pathogenically-relevant cell types, and this deficiency is due to DNA hypermethylation in FRDA. Therefore, frataxin-E is another transcriptionally dysregulated product of the *FXN* gene in FRDA. These observations redefine the detrimental effects of repeat-induced epigenetic silencing in FRDA, and warrant serious consideration of the role of frataxin E deficiency in FRDA. We posit that *FXN* gene reactivation and replacement therapies, currently in pre/early clinical development, should consider replenishing this FRDA-specific deficiency.

## Results

### FXN-E transcript originates in intron 1 of the FXN gene and is predicted to encode frataxin-E

**Figure 1:**
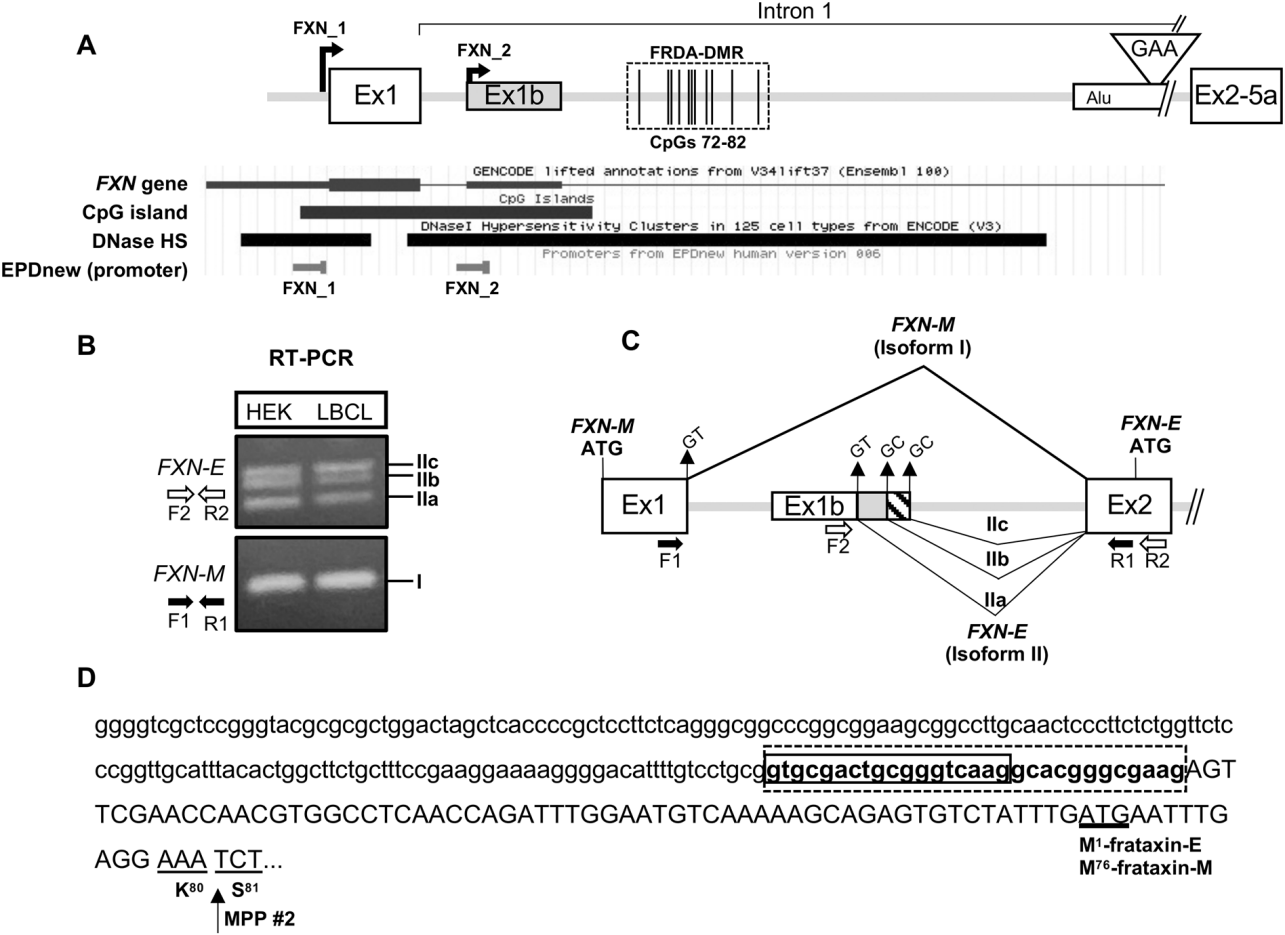
Frataxin-E is encoded by the *FXN-E* transcript that originates in intron 1 of the *FXN* gene, and its deficiency is related to the expanded GAA triplet-repeat in FRDA. (**A**) Schematic of the proximal portion of the *FXN* gene, showing the expanded GAA triplet-repeat (triangle), and the FRDA-DMR in intron 1 (dashed box; vertical lines represent the 11 CpGs, numbered 72–82). Basic gene set from GENCODE shows an alternate first exon in intron 1 of the *FXN* gene (Ex1b; located close to the FRDA-DMR), coincident with a DNase hypersensitive site (HS) and a curated promoter, per EPDnew (*FXN*_2), which is distinct from the canonical *FXN* promoter upstream of Exon 1 (*FXN*_1). (**B**) RT-PCR with a forward primer in Ex1b (F2) and reverse primer in Ex2 (R2) showing three spliceforms (IIa, IIb, and IIc). RT-PCR with a forward primer in Ex1 (F1) and reverse primer in Ex2 (R1) showing the canonical *FXN* transcript formed by splicing Exon 1 and 2. Note: Uncropped gel images are included in Fig. S14. (**C**) *FXN-E* is formed via splicing of Ex1b to Ex2 via three alternate splice donor sites to form three spliceforms (IIa, IIb, and IIc), as shown. Isoforms IIb and IIc contain an extra 18 (gray) or 30 (hatched) nucleotides of intron 1 sequence, respectively, at the 3′ end of Ex1b. Isoforms IIb and IIc use the uncommon “GC” splice donor. Isoforms IIa, IIb, and IIc are together referred to as *FXN-E*. *FXN-M*, which encodes the mitochondrial form of frataxin, is shown. (**D**) All isoforms of *FXN-E* are predicted to encode the extra-mitochondrial isoform, frataxin-E. Sequence of Ex1b (lowercase) is shown spliced to exon 2 (uppercase). The solid and dashed boxes mark the + 18 and + 30 nucleotides in isoforms IIb and IIc. The ATG initiation codon, M^76^ for frataxin-M and M^1^ for frataxin-E (underlined) is shown. The cleavage site in the second step of processing via mitochondrial processing peptidase (MPP #2) is marked by an arrow, hence frataxin-E lacks most of the mitochondrial targeting sequence.

The canonical product of the *FXN* gene, the *FXN-M* transcript, originates upstream of coding exon 1 (FXN_1; Fig. 1A)^6^. Its mature form, comprised of exons 1–5a, encodes mitochondrial frataxin (frataxin-M), which is required for efficient Fe-S cluster biogenesis^31^. Much of the mitochondrial targeting sequence is encoded by exon 1. Xia et al^32^ reported an alternate form of the *FXN* transcript that originates in intron 1 via non-coding exon 1b (Ex1b; Fig. 1A). We found that this corresponds with an annotated exon in GENCODE (71,650,949–71,651,103 per GRCh37/hg19), an overlapping DNase hypersensitive site, and a distinct experimentally-validated promoter per EPDnew (FXN_2; Fig. 1A). The promoter for the *FXN-M* transcript (FXN_1), located upstream of exon 1, and the promoter upstream of exon 1b (FXN_2) map within the same CpG island that also encompasses exon 1 and exon 1b (Fig. 1A). Analysis of a 10 × Genomics single-cell ATAC‐seq dataset from a healthy donor showed that the Ensembl-defined “*FXN* promoter” contains two discrete sites of open chromatin, in *cis* (Fig. S1), thus further supporting the existence of an independent promoter in intron 1. RNAseq data per EPDnew indicate that FXN_2 is expressed in the central nervous system and a few other cell types, albeit at lower levels and with a less-constrained transcription start site compared with FXN_1 (Fig. S2). RT-PCR analysis using HEK293T cells and a non-FRDA lymphoblastoid cell line showed that while exon 1 splices to exon 2 to form the *FXN-M* transcript (isoform I; which does not contain any exon 1b sequence), exon 1b independently splices to exon 2 (isoform II) via three alternate splice donor sites, denoted as IIa, IIb and IIc (Fig. 1B,C; note: a rare isoform, IId, identified only in lymphoblasts, contained an additional non-coding exon [exon 1c], spliced between exon 1b and exon 2 [Fig. S3]). Whereas isoform IIa uses the canonical “GT” splice donor sequence, isoforms IIb and IIc use the non-canonical “GC” splice donor sequence (Fig. 1C). The predicted translational initiation codon for all variants of isoform II (including IId; Fig. S3) is located within exon 2, and corresponds to the methionine at position 76 in frataxin-M (Fig. 1D). Thus, isoform II is predicted to encode frataxin-E (amino acids 76 to 210), which does not contain most of the mitochondrial targeting sequence found in frataxin-M (Fig. 1D; henceforth, isoforms IIa, IIb, and IIc are collectively referred to in the singular, as the “*FXN-E”* transcript). Altogether, these data indicate that frataxin-E is encoded by the *FXN-E* transcript, which originates in intron 1 of the *FXN* gene, and is distinct from the *FXN-M* transcript that codes for frataxin-M.

### Frataxin-E protein is deficient in FRDA

**Figure 2:**
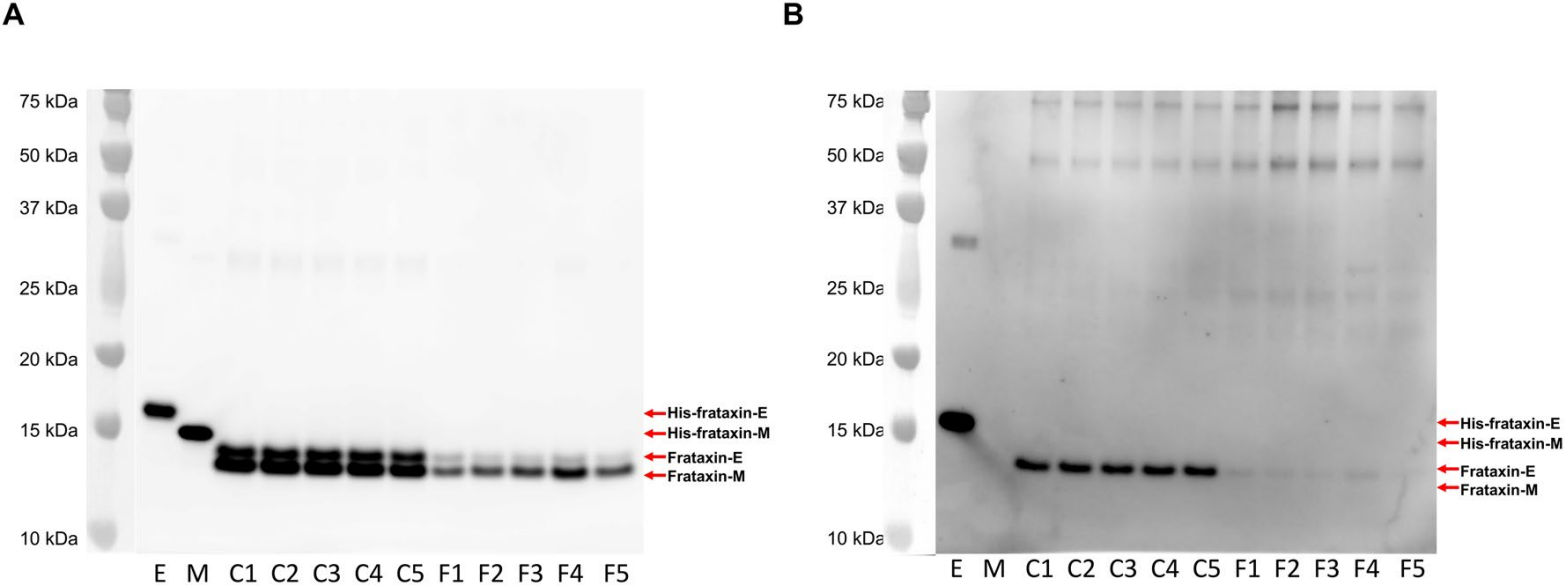
Frataxin-E is deficient in FRDA. Western blot analysis utilizing (**A**) a commercial anti-frataxin monoclonal antibody and (**B**) a specific anti-frataxin-E monoclonal antibody. Blood samples from n = 5 non-FRDA controls (C1 to C5; 0.3 mL) and n = 5 FRDA patients (F1 to F5; 0.5 mL) were immunopurified with anti-frataxin antibody and analyzed via western blot. Lanes “E” and “M” show results for histidine-tagged/purified frataxin-E and frataxin-M protein, respectively. Arrows and labels (right-hand side) show location of proteins of interest. Although there is a small difference in size (Δ 685 Da) between endogenous frataxin-E (MW = 14,953 Da) and frataxin-M (14,268 Da), these proteins were resolved on PAGE using MES and SDS as the running buffer. Frataxin-E and frataxin M together with their His-tagged standards were detected by the anti-frataxin antibody; whereas, only frataxin-E and His-frataxin E were detected by the anti-frataxin-E mAb. Protein plus protein dual color standards were run on each gel and visualized in black and white by the ImageQuant LAS 4000 camera. Western blots of the frataxin proteins were visualized separately by the camera using the SuperSignal West femto luminol enhancement reagent. The two separate images were then combined.

Blood samples (n = 5 non-FRDA controls, and n = 5 FRDA patients) were purified by immunoprecipitation (IP) using a commercial anti-human frataxin mAb and further analyzed by western blot. The presence of frataxin-M in the non-FRDA and FRDA blood samples was confirmed using the commercial anti-human frataxin mAb which detected both a His-frataxin-E standard and a His-frataxin-M standard (Fig. 2A). Endogenous frataxin-E (MW = 14,953 Da) and endogenous frataxin-M (MW = 14,268 Da) isoforms were separated by PAGE using MES with SDS as the running buffer, although they only differ in mass by 685 Da. There was clearly a deficiency of both isoforms in blood samples from FRDA patients compared to non-FRDA controls (Fig. 2A).

To determine if frataxin-E protein was deficient in FRDA, the IP purified blood samples were analyzed by western blot using a specific anti-human frataxin-E mAb generated in house. The anti-frataxin-E mAb detected a His-frataxin-E standard with < 5% cross-reaction to a His-frataxin-M standard (Fig. 2B) demonstrating specificity. Frataxin-E was hardly detectable in blood samples from FRDA patients and severely deficient compared to blood samples from non-FRDA controls (Fig. 2B).

### Frataxin-E deficiency correlates with the length of the expanded GAA triplet-repeat

**Figure 3:**
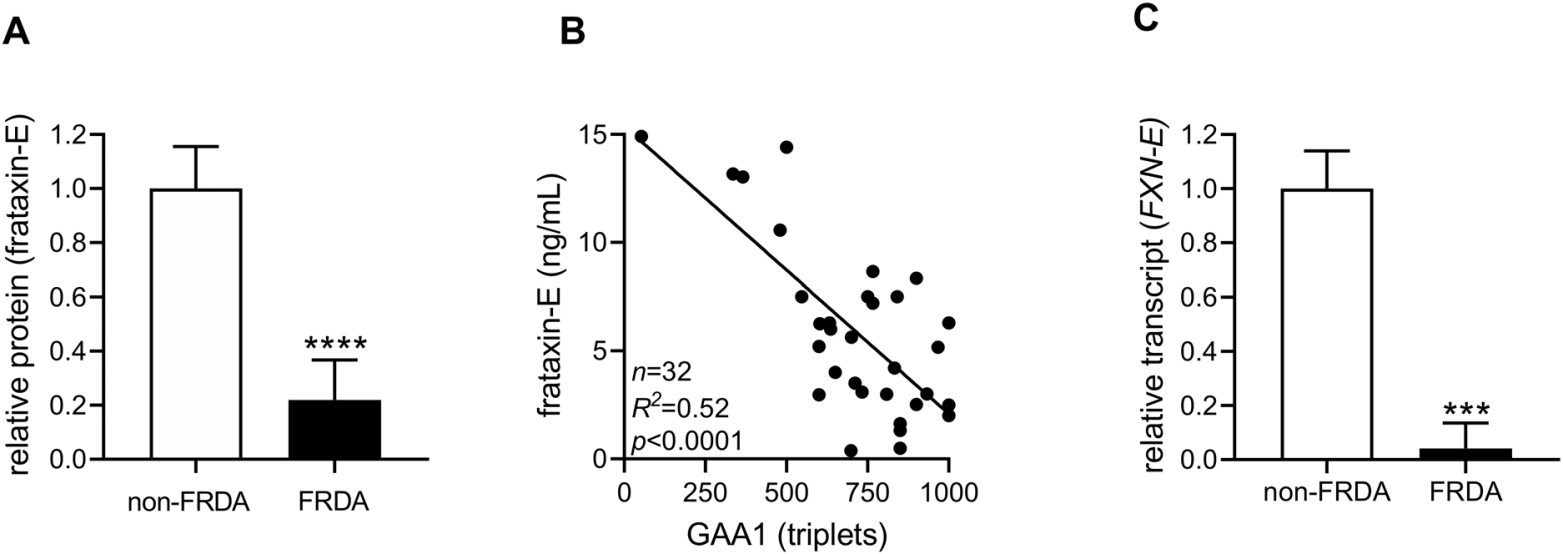
Deficiency of frataxin-E correlates with the length of the expanded GAA triplet repeat. (**A**) Frataxin-E protein in erythrocytes is deficient in FRDA (FRDA [n = 32] vs non-FRDA [n = 11]; mean ± SD; two-tailed unpaired *t* test, *p* < 0.0001, t = 14.86, *df* = 41). (**B**) Frataxin E protein is inversely correlated with the shorter of the two expanded GAA alleles (GAA1) in FRDA. (**C**) Deficiency of *FXN-E* transcript in FRDA lymphoblastoid cells (FRDA [n = 4] vs non-FRDA [n = 2]; mean ± SD; two-tailed, unpaired *t* test, *p* = 0.0001, t = 10.98, *df* = 5).

A clear relationship exists between the canonical frataxin-M protein and the length of the shorter of the two GAA repeat expansions (GAA1) in FRDA. Determining if a similar relationship exists with frataxin-E and GAA1 required a highly quantitative and accurate method for quantifying frataxin-E. Blood samples from a series of 32 FRDA patients (with a representative distribution of GAA repeat lengths^4,33^; Table S1) were examined for correlation between GAA repeat length and frataxin-E deficiency using a highly quantitative and previously validated LC–MS assay^30^. Frataxin-E was measured in erythrocytes (where it is known to be expressed, and because they lack mitochondria) and frataxin-M and GAA repeat lengths were measured in PBMCs from the same venous blood sample. Similar to the western blot results, both frataxin-E and frataxin-M were found to be deficient in FRDA patients with the LC–MS assay (n = 32 vs. n = 11 non-FRDA controls; Fig. 3A and Fig. S4A), and the deficiency was significantly correlated with the length of GAA1, which accounted for approximately half the variability (Fig. 3B and Fig. S4B). Whereas the relationship of frataxin-M deficiency with the expanded GAA repeat is well established, these data also suggest a direct relationship between the expanded GAA repeat and frataxin-E deficiency in FRDA. Consistent with previous observations^20,26,27,34^, the GAA2 allele did not correlate with either frataxin-E or frataxin-M levels (Fig. S5A,B). *FXN-E* transcript was highly deficient in patient-derived lymphoblastoid cell lines compared with non-FRDA controls (< 5% of non-FRDA; *t* test *p* = 0.0001; Fig. 3C), but the levels were unfortunately too low in lymphoblastoid cell lines (and essentially undetectable in FRDA patient PBMCs and whole blood) to permit a similar quantitative analysis for correlation with the expanded GAA repeat. As expected, the *FXN-M* transcript was also deficient in the same cell lines, but its deficiency was less severe compared with that of *FXN-E* (Fig. S4C).

### Deficiency of frataxin-E correlates with FXN DNA hypermethylation in FRDA

**Figure 4:**
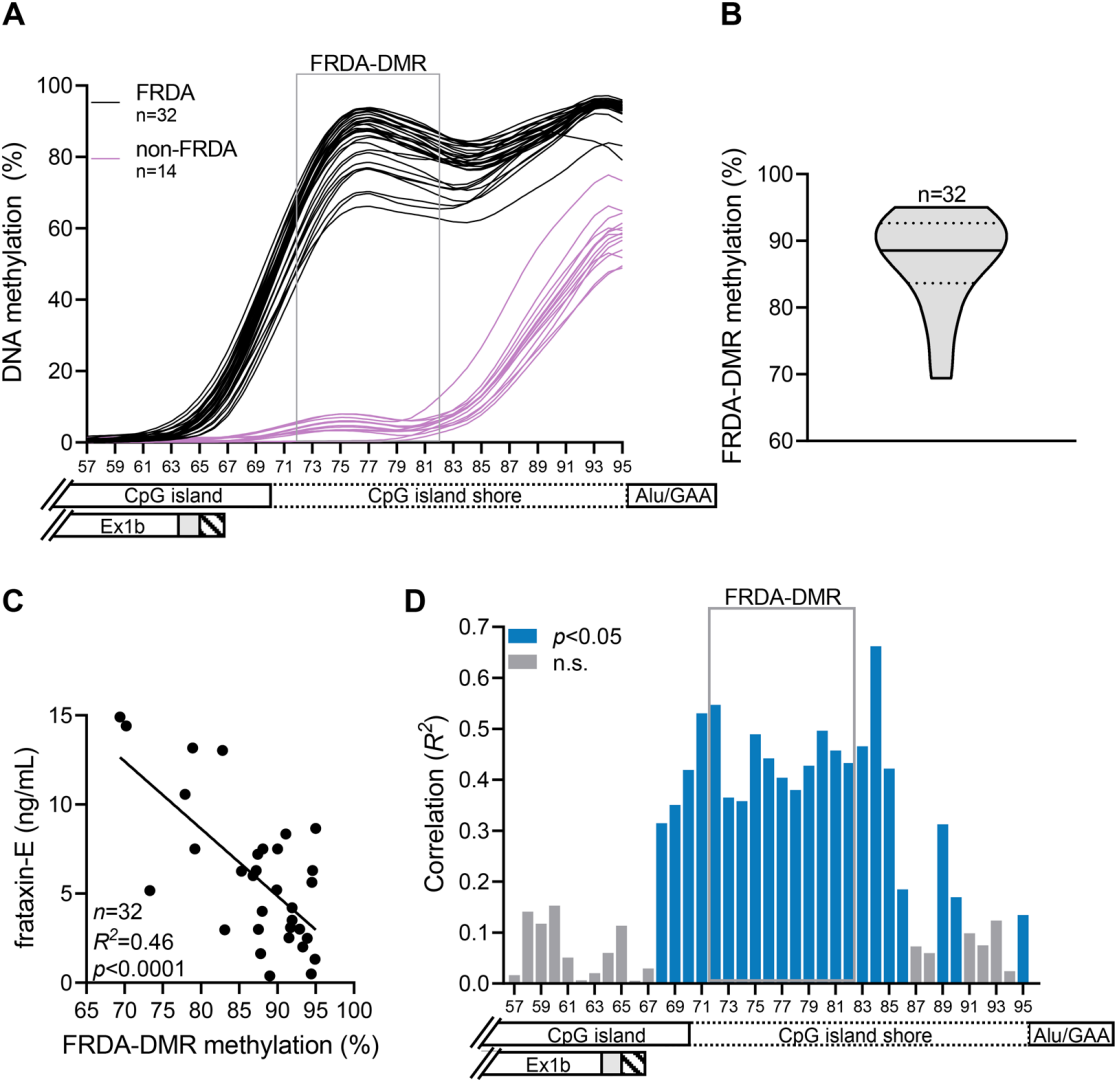
Deficiency of frataxin-E correlates with *FXN* DNA hypermethylation in FRDA. (**A**) DNA methylation is plotted as a percentage of 1000 sequencing reads, at each of 39 CpG dinucleotides (numbered 57–95) that map between the 3′ end of the CpG island and the Alu repeat element that contains the expanded GAA repeat. The data are displayed as LOWESS trendlines for FRDA (n = 32; black lines) and non-FRDA controls (n = 14; purple lines). The FRDA-DMR is outlined with a gray box, and the relative locations of the CpG island, CpG island shore, Alu/GAA, and Ex1b are indicated. (**B**) FRDA-DMR hypermethylation among the FRDA patients in our cohort (n = 32); the solid and dotted lines within the violin plot indicate median and 25th/75th percentiles, respectively. (**C**) Frataxin-E protein is inversely correlated with FRDA-DMR methylation. (**D**) Bar graph showing R^2^ values (Pearson) for correlation of frataxin-E levels in the 32 patients with methylation at each of the 39 CpG sites. Note the clustering of CpG sites showing significant correlation (blue bars; with Benjamini and Hochberg correction for multiple comparisons) in the FRDA-DMR (gray box). Gray bars indicate correlations that are not significant (n.s.). The relative locations of the CpG island, CpG island shore, Alu/GAA, and Ex1b are displayed below the graph.

The expanded GAA triplet-repeat in FRDA induces DNA hypermethylation in intron 1, in a region with 11 contiguous CpG sites, which forms a defined FRDA-specific differentially methylated region (FRDA-DMR) located immediately downstream from the *FXN* CpG island (Fig. 1A)^22^. Bisulfite deep sequencing (n = 1000 sequence reads per CpG) in PBMCs from our cohort of 32 FRDA patients was used to determine the methylation status at all 39 CpG sites (numbered 57 to 95 per Rodden et al.^22^ along the X-axis in Fig. 4A) that span the region of intron 1 from the 3’ end of the CpG island (and exon 1b; Fig. 4A) to the expanded GAA triplet-repeat. This showed the typical hypermethylation of the FRDA-DMR, which ranged from 69 to 95% in FRDA patients (n = 32; Fig. 4A,B), compared with 4% in non-FRDA controls (n = 14; *p* < 0.0001; Fig. 4A,B; Fig. S6). Frataxin-E protein level in FRDA was inversely correlated with methylation in the FRDA-DMR (Fig. 4C; interestingly, this correlation was stronger than for frataxin-M, Fig. S7). Almost all patients with frataxin-E levels of < 5 ng/ml had > 85% methylation, and the few patients with > 10 ng/ml had < 85% methylation (Fig. 4C). Methylation levels at each of the 39 CpG sites were individually assessed for correlation with frataxin-E levels in FRDA. This unbiased approach revealed that frataxin-E levels correlated specifically with all of the CpG sites within the FRDA-DMR (numbered 72–82; Fig. 4D), and the correlation weakened and eventually disappeared at CpGs away from the FRDA-DMR, thus spatially localizing the correlation within the FRDA-DMR. These data tie frataxin-E deficiency to the downstream epigenetic consequences of the expanded GAA triplet-repeat, and suggest a causal relationship with FRDA-specific DNA hypermethylation.

### FXN-E transcript is deficient in pathologically relevant cell types in FRDA

**Figure 5:**
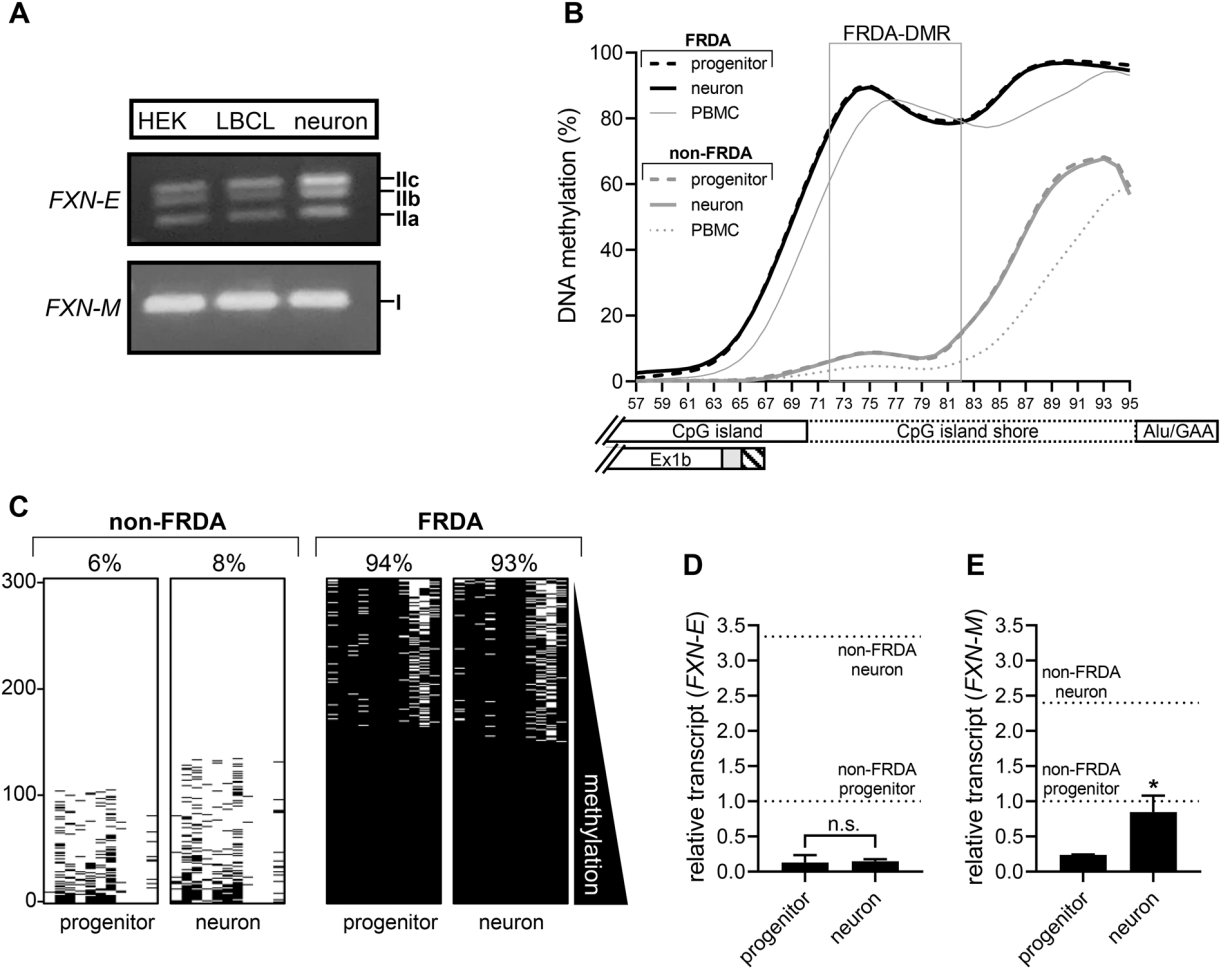
FRDA-DMR hypermethylation and deficiency of *FXN-E* transcript in iPS-derived proprioceptive neurons in FRDA. (**A**) Proprioceptive neurons derived from non-FRDA control iPS lines express *FXN-M* (isoform I) and *FXN-E* (isoforms IIa, IIb, and IIc). Note: Uncropped gel images are included in Fig. S14. (**B**) DNA methylation is plotted as a percentage of 1000 sequencing reads, at each of 39 CpG dinucleotides (numbered 57–95) that map between the 3′ end of the CpG island and the Alu repeat element that contains the expanded GAA repeat. Data are displayed as LOWESS trendlines for FRDA (neurons [n = 3]; neuronal progenitors [n = 3]; PBMCs [n = 32]) and non FRDA controls (neurons [n = 1]; neuronal progenitors [n = 1]; PBMCs [n = 14]). The FRDA-DMR is outlined with a gray box, and the relative locations of the CpG island, CpG island shore, Alu/GAA, and Ex1b are indicated. (**C**) FRDA-DMR methylation assayed as a single amplicon to test each of the n = 11 CpG dinucleotides in *cis* displayed as n = 300 sequencing reads stacked vertically for both neuronal progenitors and proprioceptive neurons from FRDA and non-FRDA controls (black dash = methylated CpG; reads are sorted with highest methylation at the bottom). (**D**) *FXN-E* and (**E**) *FXN-M* transcript levels in non-FRDA neuronal progenitors (n = 1) and proprioceptive neurons (n = 1) are shown as dotted horizontal lines. Transcript levels in FRDA neuronal progenitors (n = 3) and proprioceptive neurons (n = 3) are shown as black bars (mean ± SD); for *FXN-E*: *p* = 0.7953, t = 0.2773, df = 4, and for *FXN-M*: *p* = 0.0107, t = 4.511, df = 4.

As opposed to the *FXN-M* transcript, which is widely expressed, initial experiments showed that *FXN-E* transcript was detectable in lymphoblastoid cell lines and HEK293T cells (Fig. 1B), but barely detectable in PBMCs and whole blood. To test if *FXN-E* is expressed in pathologically relevant cell types in FRDA, we examined iPS-derived proprioceptive neurons and neuronal progenitors from a non-FRDA control subject. This showed the same three predominant spliceforms of *FXN-E* formed by alternate splicing of exon 1b with exon 2 (isoforms IIa, IIb, IIc) as were detected in HEK293T cells and lymphoblastoid cells (Fig. 5A; Fig. S8). Bisulfite deep sequencing of iPS-derived proprioceptive neurons and neuronal progenitors (n = 3 FRDA patients; n = 1000 sequence reads per CpG) showed FRDA-specific DNA hypermethylation in intron 1, similar to what we observed in FRDA PBMCs (Fig. 5B). Deep sequencing of the FRDA-DMR as a single amplicon (n = 1000 sequence reads) revealed FRDA-specific hypermethylation (> 90%) in proprioceptive neurons and progenitors, similar to FRDA PBMCs and LBCLs (Fig. 5C; Fig S9A-D; note: methylation panels show only 300 of 1000 sequence reads analyzed). Quantitative RT-PCR showed that both *FXN-E* and *FXN-M* transcripts increase 2 to threefold upon differentiation of non-FRDA neuronal progenitors into proprioceptive neurons (Fig. 5D,E). In FRDA, *FXN-E* is highly deficient in both progenitors and proprioceptive neurons, and no increase was seen in transcript level upon neuronal differentiation (Fig. 5D). In contrast, the *FXN-M* transcript, while deficient in FRDA progenitors and proprioceptive neurons, did show a response, albeit a muted one of increasing transcript level upon neuronal differentiation (thus achieving the level of *FXN-M* transcript seen in the non-FRDA neuronal progenitor cells; Fig. 5E). Somatic instability of the expanded GAA repeat was ruled out as a variable, as both expanded repeats in all three FRDA lines remained unchanged upon neuronal differentiation (Fig. S10; Table S2).

**Figure 6:**
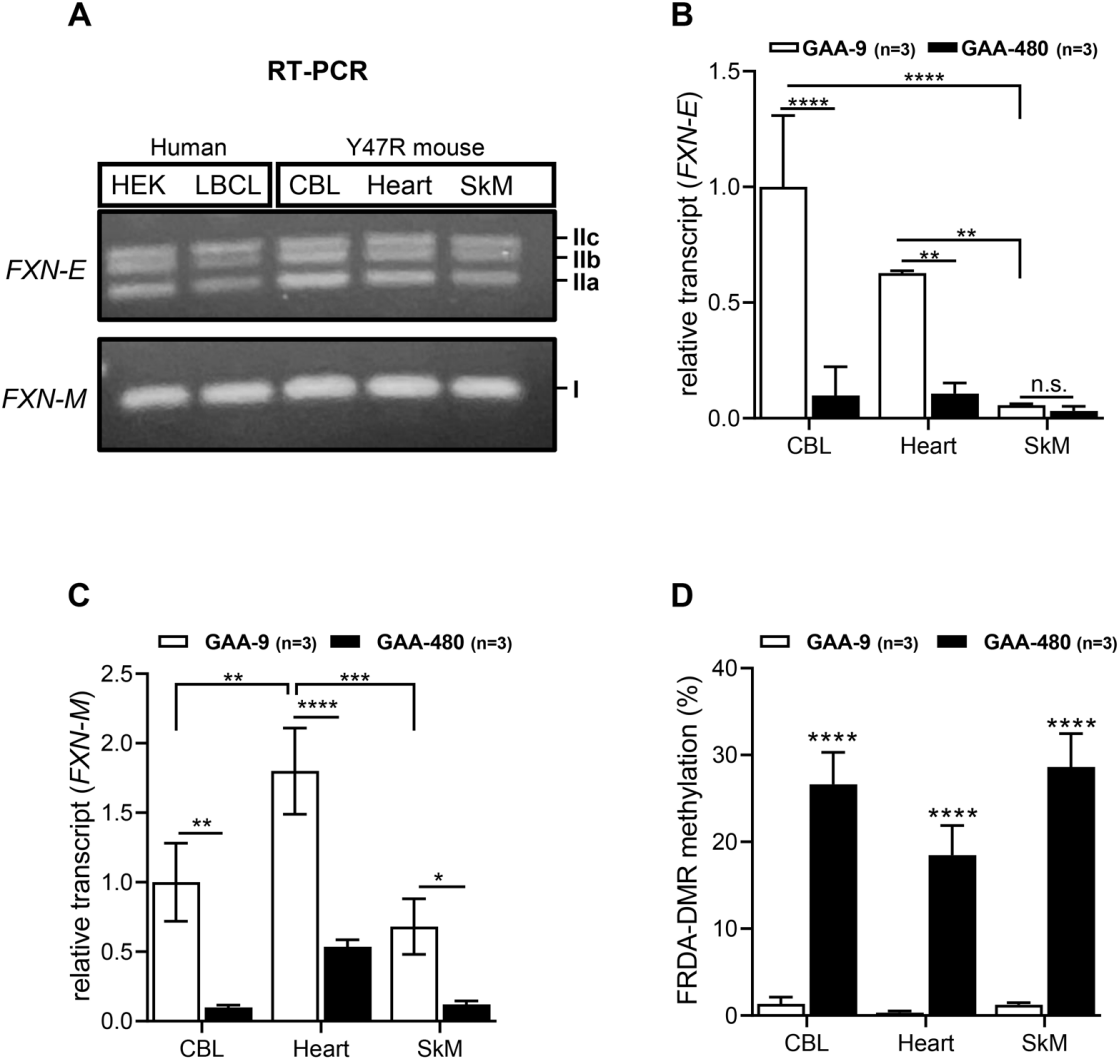
FRDA-DMR hypermethylation and deficiency of *FXN-E* transcript in tissues from the YG8sR humanized mouse model of FRDA. (**A**) Cerebellum (CBL), heart, and skeletal muscle (SkM) from the non-FRDA control mouse (Y47R; with 9 GAA triplets) express *FXN-M* (isoform I) and *FXN-E* isoforms (IIa, IIb, and IIc). Note: Uncropped gel images are included in Fig. S14. **(B**) Relative *FXN-E* transcript levels in CBL, heart, and SkM in Y47R mice (n = 3) and in the FRDA mouse model (YG8sR; with 480 GAA repeats; n = 3). (**C**) Relative *FXN-M* transcript levels in CBL, heart, and SkM in Y47R (n = 3) and YG8sR (n = 3) mice. (**D**) FRDA-DMR methylation levels in CBL, heart, and SkM from the Y47R (GAA-9; n = 3) and YG8sR (GAA-480; n = 3) mice. All graphs show mean ± SD; 2way ANOVA with Tukey’s multiple comparisons; *p < 0.05, **p < 0.01, ***p < 0.001, ****p < 0.0001, n.s. = not significant.

We next analyzed cerebellum, heart and skeletal muscle from a humanized mouse model, wherein the human *FXN* transgene, containing either 9 (Y47R) or 480 (YG8sR) GAA triplets, rescues the lethality of the *FXN*-null mouse^35^. RT-PCR of tissues from the Y47R mouse showed the same three predominant spliceforms of *FXN-E* formed by alternate splicing of exon 1b to exon 2 (isoforms IIa, IIb, IIc; Fig. 6A). In the Y47R mouse, *FXN-E* was found to be expressed in the cerebellum and heart but was seen at very low levels in skeletal muscle (Fig. 6B), which contrasted with *FXN-M*, which was detectable in all three tissues, and expressed at a relatively high level in the heart (Fig. 6C). Both *FXN-E* and *FXN-M* transcripts were deficient in all tissues in the YG8sR mouse (GAA-480), although the deficiency of *FXN-M* transcript was less pronounced in the heart (Fig. 6B,C). The YG8sR mouse clearly showed increased DNA methylation in the FRDA-DMR in all tissues, but it was notable for being rather low (20–30% methylation, with somewhat lower levels in the heart) compared with what is seen in human cell types with expanded GAA repeats (Fig. 6D). There were no obvious differences in the level of FRDA-DMR methylation in male versus female mice, and in young (1 month) versus older (12 month) mice (Fig. S11A-C). Somatic instability of the expanded GAA repeat was ruled out as an explanation for the variability in levels of transcript or DNA methylation, as the expanded repeat remained mostly unchanged in all three tissues, across different mice (Fig. S12; note: slight instability was noted in the cerebellum, consistent with previous studies^36–38^).

### DNA hypermethylation of the FRDA-DMR silences FXN-E

**Figure 7:**
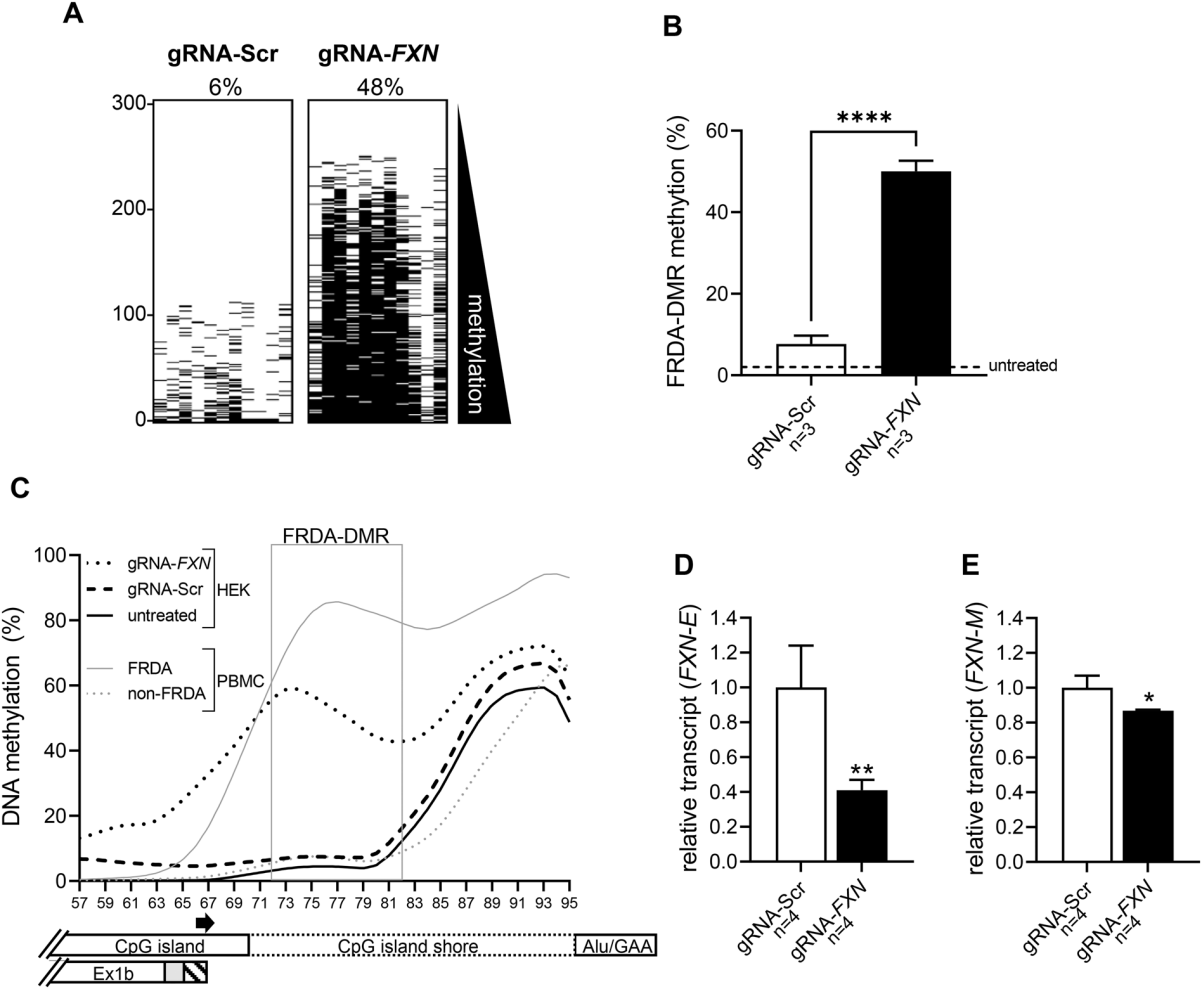
DNA hypermethylation of the FRDA-DMR silences *FXN-E*. (**A**) FRDA-DMR methylation assayed as a single amplicon to test each of the n = 11 CpG dinucleotides in *cis* displayed as n = 300 sequencing reads stacked vertically for HEK293T cells transfected with dCas9-DNMT3A targeted to intron 1 of *FXN* via a guide RNA (gRNA-*FXN*) or with a scramble guide used as a negative control (gRNA-Scr). Black dash = methylated CpG; reads are sorted with highest methylation at the bottom and percent methylation in the FRDA-DMR is displayed above the respective panels. (**B**) FRDA-DMR methylation (mean ± SD) for n = 3 experiments (****p < 0.0001, t = 21.78, *df* = 4). FRDA-DMR methylation for untreated HEK293T cells is shown as a dashed line for comparison. (**C**) DNA methylation is plotted as a percentage of 1000 sequencing reads, at each of 39 CpG dinucleotides (numbered 57–95) that map between the 3′ end of the CpG island and the Alu repeat element that contains the expanded GAA repeat. Data are displayed as LOWESS trendlines for HEK293T cells either untreated or treated with dCas9-DNMT3A+ gRNA-*FXN* (to target the FRDA-DMR) or dCas9- DNMT3A+ gRNA-Scr (negative/scramble control). Non-FRDA and FRDA PBMCs are shown for comparison. The FRDA-DMR is outlined with a gray box, and the relative locations of the CpG island, CpG island shore, Alu/GAA, and Ex1b are indicated. The location of the gRNA is indicated by the rightward pointing arrow alongside the X-axis. (**D**) Relative *FXN-E* transcript levels and (**E**) Relative *FXN-M* transcript levels for n = 4 transfections with either dCas9-DNMT3A + gRNA-*FXN* (to target the FRDA-DMR; black bars) or dCas9-DNMT3A+ gRNA-Scr (negative/scramble control; white bars). Transcript levels are shown as mean ± SD; Two-tailed, unpaired *t* test; for *FXN-E*: *p* = 0.0038, t = 4.579, *df* = 6, and for *FXN-M*: *p* = 0.0106, t = 3.656, *df* = 6. Note: despite the statistical significance of *FXN-M*, the magnitude of change is not considered biologically significant, and *FXN-M* was not significantly altered in another independent experiment (see Fig. S13).

To explore a causal relationship between DNA methylation and *FXN-E* transcriptional deficiency, a modified CRISPR system was used to modulate DNA methylation specifically in the FRDA-DMR. Catalytically inactive Cas9 tethered to DNA methyltransferase 3A^39^ was targeted to intron 1 of the *FXN* gene via a guide RNA (gRNA) designed to target the FRDA-DMR. In HEK293T cells, FRDA-DMR methylation increased from 8% (6–10%) with a scramble gRNA control to 50% (47–53%) with the targeting gRNA (*p* < 0.0001; Fig. 7A,B; the location of the targeting gRNA is depicted by an arrow alongside the X-axis in Fig. 7C). Methylation analysis of all 39 CpG sites from the 3’ end of the CpG island to the GAA repeat showed that the normal (non-FRDA) pattern of methylation, i.e., very low in the FRDA-DMR and rising slightly towards the GAA repeat, was seen in untreated and scramble-treated HEK293T cells (Fig. 7C, which was comparable to non-FRDA PBMCs). The *FXN* targeting gRNA produced a level of methylation in the FRDA-DMR that was approximately two-thirds of the level seen in PBMCs from a heterogeneous group of FRDA patients (Fig. 7C; FRDA PBMC data are depicted as a composite of the 32 patients in Fig. 3A), and it was mostly located within the FRDA-DMR. Despite this modest level of CRISPR-mediated DNA methylation in the FRDA-DMR, *FXN-E* transcript was significantly suppressed (Fig. 7D). Given that HEK293T cells do not have the expanded GAA triplet-repeat^22^, this indicates that DNA hypermethylation of the FRDA-DMR is sufficient to induce *FXN-E* transcriptional deficiency. In contrast, the *FXN-M* transcript showed a very slight trend towards deficiency (Fig. 7E). These results, i.e., convincing suppression of *FXN-E* but only a slight trend for *FXN-M* transcript, were confirmed in another complete experiment, also performed in triplicate (Fig. S13A,B).

## Discussion

The major product of the human *FXN* gene is the *FXN-M* transcript (which codes for 210 amino acids) and frataxin-M protein, which consists of residues 81-210 and is localized in the mitochondria^40,41^. The terms extramitochondrial and cytosolic frataxin have been used over the years^42–44^ to suggest a possible cytoplasmic localization and functional role for the form of frataxin containing residues 81–210. Frataxin-E, on the other hand, is a distinct isoform consisting of residues 76–210 (with an acetylated N-terminus) and is found at relatively high levels in erythrocytes^30^, and is extramitochondrial because it does not possess a mitochondrial targeting sequence. In addition, erythrocytes lose their mitochondria during maturation. In our original study, we showed the presence of isoform-E protein in erythrocytes and whole blood samples from healthy subjects using PAGE and western blot analysis with a mouse anti-human frataxin mAb^30^. We have now used a specific mouse anti-frataxin-E mAb to show that isoform E levels are lower in whole blood samples from FRDA patients compared to non-FRDA controls (Fig. 2B). Our specific quantitative stable isotope dilution IP–LC–MS assay showed that frataxin-E levels were threefold higher in erythrocytes compared with frataxin-M in PBMCs/platelets^30^. In erythrocyte samples, only 1.3–2.0% of total frataxin is frataxin-M, with the likely sources being reticulocytes and/or low levels of non-erythrocytic cells. While the function of frataxin-E is not yet well defined, it is expressed at levels that suggest a physiological function in the erythrocytic lineage. Moreover, the transcript that codes for frataxin-E (*FXN-E)*, is detected at relatively high levels in the cerebellum and heart, and early studies suggest that the cytosolic product expressed by this transcript plays a role in modulating mitochondrial function^32,45^.

We focused our study on frataxin-E and *FXN-E* precisely because of its origin in intron 1, i.e., in close proximity to the site of maximal DNA methylation in FRDA. The FRDA-DMR, which shows GAA repeat-dependent hypermethylation, is predictive of age of onset in FRDA^22^, and *FXN* gene reactivation^46^, and here we show that it is also involved in regulating expression of frataxin-E. A caveat of our study is that in patient-derived blood samples we measured frataxin-E protein in erythrocytes, but DNA methylation (and GAA repeat length) was necessarily assayed in nucleated blood cells (PBMCs). How erythrocytes in FRDA patients end up with deficiency of frataxin-E is unclear. However, a reasonable explanation is that it originated in a nucleated erythrocytic precursor(s) that was susceptible to the epigenetic silencing signals seen in FRDA, thus ultimately resulting in partitioning of deficient quantities of frataxin-E in mature erythrocytes.

While DNA methylation in the FRDA-DMR clearly suppressed the *FXN-E* transcript, the suppression of *FXN-M* transcript was not very convincing. The reason for this is unclear, but it may be due to one or more of the following reasons: the relatively modest level of methylation induced by the CRISPR strategy; the relative proximity of the FRDA-DMR to the FXN_2 promoter; and that DNA methylation per se may be insufficient to silence the *FXN-M* transcript. FXN_2 is a distinct promoter from FXN_1, and the transcripts they produce show tissue-specific differences in expression, so while there may be some co-regulation (for instance, on account of sharing the same CpG island), it would not be particularly surprising that they are differentially regulated by DNA methylation in the FRDA-DMR.

As not much is known about the function of frataxin-E, it is difficult to attribute specific phenotypic aspects of the disease to its deficiency. Extra-mitochondrial frataxin seems to play a role in mitochondrial function^32,43–45^, and in vitro studies of a cytosolic version of frataxin showed that it was capable of interacting with the Fe-S cluster assembly machinery and was protective against oxidative damage of cytosolic aconitase^32^. Carefully designed studies will be needed to determine the function of the endogenously expressed version of this isoform separately from mitochondrial frataxin. The vast majority of patients (~ 95%) are homozygous for the expanded GAA repeat^6^, and the remaining patients have one expanded allele and another pathogenic variant in the other *FXN* allele^47–51^. Among these compound heterozygous individuals, those with some missense variants and those with truncating variants located upstream of exon 1b (e.g. changes affecting the translation initiation codon of *FXN-M*) are likely to have at least half the normal level of frataxin-E. Genotype–phenotype correlations in FRDA have so far not considered such differential effects on frataxin-E expression, and we suggest that doing so could be a potentially useful way to delineate the phenotypic contribution of this lesser known product of the *FXN* gene.

Given that expression is detectable in cell types and tissues that are relevant to FRDA pathophysiology, it is imperative to uncover any potential deleterious effects of frataxin-E deficiency in FRDA. This is critical because gene and protein replacement therapies are actively being developed with the specific goal of replenishing frataxin-M^52–54^, i.e., inadvertently not addressing the deficiency of frataxin-E. It is noteworthy that gene therapy constructs containing varying lengths of intron 1 sequence have been designed^55^, and these could potentially deliver both isoforms of frataxin. Indeed, *FXN* gene reactivation strategies, also being developed^10,28,56,57^, may have an added advantage of utilizing the endogenous gene regulatory elements that could permit reactivation of both isoforms.

## Materials and methods

### Materials

Protein G Dynabeads (catalog 10009D), NuPAGE 12% bis–Tris protein gels (catalog NP0341PK2), NuPAGE 3-(N-morpholino)ethanesulfonic acid (MES) sodium dodecyl sulfate (SDS) running buffer (catalog NP0002), NuPAGE lithium dodecyl sulfate (LDS) sample buffer (4X, catalog NP0007), β-mercaptoethanol (BME, catalog 21985023), iBlot 2 gel transfer device (IB21001), iBlot 2 transfer stacks, nitrocellulose (catalog IB23001), SuperSignal West femto stable peroxide buffer (catalog 1856190), SuperSignal West femto luminol enhancer solution (catalog 1856189), aqueous 0.2M triethanolamine, pH 8.0 (catalog J63793), dimethyl pimelimidate (DMP) dihydrochloride (catalog 21667), UltraPure Tris-HCI buffer, pH 7.5 (catalog 15567027), Ultrapure ethylenediaminetetraacetic acid (EDTA) dihydrate (catalog 15575020), and Optima water (catalog W6500) were from ThermoFisher Scientific (Waltham, MA). Precision plus protein dual color standards were from Bio-Rad (Hercules, California). DL-dithiothreitol (DTT) (catalog 43815), Triton X-100 (catalog X100RS-25G), Tween-20 (catalog P1379), Eppendorf LoBind microcentrifuge tubes (Catalog EP0030108051), Roche Mini EDTA-free, Easypack protease inhibitor cocktail tablets (catalog 11836170,001), and (Dulbecco's Modified Eagle Medium (DMEM) were from MilliporeSigma (Billerica, MA). One tablet of protease inhibitor was dissolved in Optima water (0.5 mL). LC grade acetonitrile and acetic acid were from Burdick and Jackson (Muskegon, MI, USA). The mouse anti-human frataxin mouse monoclonal antibody (mAb) (catalog 7A11, Ab 113,691) was from Abcam (Cambridge, MA). Specific mouse anti-human frataxin-E monoclonal antibody (mAb) was prepared in collaboration with GenScript (Piscataway, NJ) from 19F1-1 hybridoma cells as described below. The secondary antibody for western blots was peroxidase-conjugated AffiniPure Goat anti-Mouse IgG, Fcy fragment specific (HRP Jackson 115-035-071).

### Study participants and samples

Lymphoblastoid cell lines were purchased from Coriell Institute for Medical Research, including those from FRDA individuals (GM16209, GM14518, GM16197, GM16204, GM16207) and non-FRDA controls (GM22647, GM22671). Blood samples were obtained from Friedreich ataxia patients with a confirmed DNA diagnosis (homozygous for the expanded GAA repeat) and from healthy donors enrolled in an ongoing natural history study of FA in accordance with the Declaration of Helsinki and IRB approval from the Children’s Hospital of Philadelphia (CHOP IRB 01-002609) and the University of Oklahoma Health Sciences Center (OUHSC IRB 8071). Informed consent was obtained from all subjects and/or legal guardians. Creation of iPS-derived proprioceptive neurons has been previously described^58^. HEK293T cells were purchased from ATCC® (CRL-11268^tm^).

### Mouse tissues

YG8sR (480 GAA repeats) and Y47R mice (9 GAA repeats) were bred, euthanized and autopsied at 1 and 12 months of age at Brunel University London (U.K.) under humane conditions in accordance with the U.K. Home Office “Animals (Scientific Procedures) Act 1986” and with approval from the Brunel University London Animals Welfare and Ethical Review Board (Project license number PPL303031). Mice were euthanized for dissection and tissue collection by a Schedule 1 method (cervical dislocation) in accordance with the UK Animals (Scientific Procedures) Act 1986. Frozen tissue samples were transported on dry ice via overnight courier service to the University of Oklahoma Health Sciences Center in Oklahoma City for analysis. All relevant information pertaining to a controlled, non-interventional study is provided in the relevant sections of Results and Methods in accordance with ARRIVE guidelines.

### Estimation of GAA triplet-repeat by long-range PCR analysis

GAA repeat lengths were measured using a long-range PCR assay (AccuStart Long Range SuperMix kit, Quantabio) with primers 104F and 629R flanking the GAA repeat in intron 1^7^.

### Characterization of the FXN-E transcript

RT-PCR products generated with primers spanning *FXN* Exon 1b – Exon 2 (F2 & R2 in Fig. 1B) were analyzed by gel electrophoresis. TOPO-cloned libraries (TOPO™ TA Cloning™ Kit; ThermoFisher Scientific) were generated and multiple clones were analyzed by Sanger sequencing to determine the exact sequence and splice junctions of exon 1b to exon 2.

### Immunopurification (IP) of whole blood

Protein G Dynabeads (5 mg, approximately 165 μL) were transferred to a clean tube and washed three times with the bead washing buffer consisting of 1 mL of phosphate-buffered saline (PBS) containing 0.02% Tween-20. The relevant mouse-anti-frataxin mAb (40 μL, 40 μg) was diluted with PBS to a final volume of 500 μL and incubated with the protein G Dynabeads at 4 °C overnight on a rotator. The antibody solution was removed and the beads washed twice for 5 min on a rotator with the cross-linking buffer of aqueous 0.2 M triethanolamine, pH 8.0 (1 mL). Fresh DMP solution was prepared by dissolving DMP dihydrochloride (13 mg) in 2 mL of the cross-linking buffer. The washed beads were incubated with the DMP solution (2 mL) at room temperature for 1 h on a rotator. The DMP solution was removed, the beads washed twice with 1 mL of quenching buffer consisting of 0.1 M ethanolamine, pH 8.5, then incubated with 1 mL of quenching buffer at room temperature on a rotator for 1 h. The quenching buffer was removed and the beads washed twice with bead washing buffer (1 mL). Whole blood (0.5 mL) was diluted with 0.75 mL of 1 mM DTT, protease inhibitor, and IP lysis buffer consisting of 50 mM Tris buffer pH 7.5, 150 mM sodium chloride, 1 mM EDTA, 0.5% Triton X-100, and 0.5% IGEPAL CA630. Samples were sonicated for 30 pulses (~ 0.5 s each) with a probe sonicator at a power of 4 then spun down at 4 °C at 16,000×*g* for 10 min. The supernatant of the DMP cross-linked beads was removed, the samples added to the beads, and incubated at 4 oC overnight on a rotator. The unbound sample was removed the beads in 1 mL beads re-suspended in the wash buffer. The beads were washed a further three times with the wash buffer, transferred to a clean 1.5 mL LoBind tube with the 3rd wash, and then washed with PBS (1 mL). After removal of the PBS, the elution buffer consisting of 100 mM acetic acid/10% acetonitrile (100 μL) was added and the beads were incubated 37 °C for 1 h at 1000 rpm in the 1.5 mL LoBind tubes. The supernatant was transferred to a de-activated tube, dried under nitrogen flow for 1 h, then re-suspended in PBS (50 μL) ready for PAGE analysis.

### Preparation of mouse anti-frataxin-E mAb

The mAb was generated in collaboration with GenScript from a frataxin-E acetylated N-terminal peptide antigen, which contained amino acids 76–85 (acetyl-MNLRKSGTLGC) linked through a C-terminal cysteine residue to keyhole limpet hemocyanin. Briefly, BALB/c mice were immunized with the frataxin-E peptide antigen, spleens from immunized animals were isolated, placed in a sterile disposable petri dish, and ground with a syringe, DMEM media was added, the B-lymphocytes washed with DMEM, and isolated by centrifugation. SP2/0 myeloma cells were then combined with the B-lymphocytes. The tubes were centrifuged, the cell pellets collected, and suspended in complete fusion media containing hypoxanthine-aminopterin-thymidine (HAT) medium to a density of 2 × 10^5^ cells/mL. The cell suspension (100 μL) was added to each well of the 96-well fusion plate coated with feeder cells and the plates incubated at 37 °C with 6% CO_2_ in a humidified incubator for 7-days. Hybridoma cell supernatants were screened after 7-days and the plates maintained after the screening. Analyses using His-frataxin-E as the antigen were conducted to identify positive clones. Two of the positive clones were sub-cloned with HT media lacking aminopterin. Plates were incubated at 37 °C with 6% CO_2_ in a humidified incubator for 7-days. The supernatants were transferred from the wells (with the cell colony) for screening after 7 days cell growth. The most specific sub-clone that was obtained (19F1-1) was expanded first in a 25 cm^2^ flask and then in 75 cm^2^ flasks containing HT media. Specificity of the mAb obtained from expansion of the 19F1-1 clone was confirmed by western blot analysis using His-frataxin-E as the antigen.

### Western blot analysis

The His-frataxin-E standard (2 ng), His-frataxin-M standard (2 ng) and a portion from each whole blood eluate (20 μL) were mixed separately with 5 μL of NuPAGE LDS sample buffer (4X) containing 8% BME. The samples were then heated to 95 °C for 10 min before loading on a 12% NuPAGE Bis–Tris protein gel. NuPAGE MES SDS buffer was used for optimal separation of proteins in the 10–30 kDa range. The gel was run under 150 V for 1.5 h until the blue dye ran to the bottom of the gel. The proteins were transferred to a nitrocellulose membrane using the iBlot 2 gel transfer device and an iBlot 2 transfer stack. The membrane was probed with either an Abcam (Ab113691) anti-human frataxin mouse mAb (diluted 1:1000 with 5% milk in PBS containing 0.1% Tween-20) or an anti-human frataxin isoform E mAb generated by GenScript (diluted 1:1000 with 5% milk in PBS containing 0.1% Tween-20). A goat anti-rabbit HRP IgG (diluted 1:5000) was used as the secondary antibody for chemiluminescence detection. Chemiluminescence was generated using a 1:1 mixture of SuperSignal West femto stable peroxide buffer and luminol enhancer solution. Western blot images were captured on an ImageQuant LAS 4000 (GE Healthcare, Piscataway, NJ).

### Quantification of frataxin-M and frataxin-E by LC–MS analysis of frataxin-derived peptides

The expression of unlabeled and stable isotope labeling by amino acids in cell culture (SILAC)-labeled mature frataxin was performed in *Escherichia coli* BL21 DE3 as described previously^30,61^. They each had GSGSLEHHHHHH carboxy-terminal His-tags. All blood samples were thawed at room temperature, and 500 µL of each sample was mixed with 750 µL NP-40 lysis buffer (150 mM NaCl, 50 mM Tris/HCl pH 7.5, 0.5% Triton X-100, 0.5% NP-40, 1 mM DTT, 1 mM EDTA) containing protease inhibitor cocktail. The same amount of SILAC-labeled mature frataxin (20 ng) was spiked in each sample as an internal standard. Samples were lysed and incubated with pre-made DMP-crosslinked anti-frataxin protein G beads for immunopurification as described previously^61^. Samples were analyzed by liquid chromatography-mass spectrometry (LC–MS) analysis as described previously^30^. For peptide quantification parallel reaction monitoring (PRM) was scheduled for 20.5 to 22.5 min for SGTLGHPGSL, 24.0 to 28.0 min for DVSFGSGVLTVKLGG, 22.00 to 24.2 min for DWTGKNWVYSH, and 21.5 to 23.5 min for DLSSLAYSGK. Data analysis for protein quantification was performed using Skyline (MacCoss Laboratory, University of Washington, Seattle, WA). The peak area ratio of each PRM transition for each unlabeled/light (L) peptide to labeled/heavy (H) peptide was calculated by the Skyline software and used for absolute quantification. Total frataxin was determined from DVSFGSGVLTVKLGG, DWTGKNWVYSH, and DLSSLAYSGK. Frataxin-M was determined from SGTLGHPGSL and frataxin-E was determined from the difference between total frataxin and frataxin-M.

### Quantitative RT-PCR

Total RNA (1 μg) was reverse transcribed using the QuantiTect^®^ reverse transcription kit (Qiagen). Transcript levels were quantified by real-time PCR with primers spanning the splice junction of *FXN* exons 3 and 4, *FXN* exons 1 and 2, or *FXN* exons 1b and 2, relative to expression of *RPL27* (for human samples) or β-actin (for mouse samples) using the ΔΔCt method. Power SYBR green PCR mastermix (Applied Biosystems) was used on a Roche LightCycler^®^ 96 System. Reaction conditions included a two-step amplification protocol of 40 cycles of 95 °C for 15 s and 62 °C for 45 s. Primer sequences for *FXN* exons 3 to 4 *RPL27*, and β-actin were as previously described^10,59,60^. Primer sequences for *FXN* Exon1-Exon2 (F1 & R1 in Fig. 1B) are F1: 5′-GCACCGACATCGATGCGACC-3′, and R1: 5′-GACATTCCAAATCTGGTTGAGG-3′ and for *FXN* Exon1b-Exon2 (F2 & R2 in Fig. 1B) are F2: 5′-AAGGAAAAGGGGACATTTTGT-3′, and R2: 5′-GTGGCCCAAAGTTCCAGATTTC-3′.

### DNA methylation analysis

The DNA methylation assay, analysis, and validation were previously described in detail^22^. Briefly, genomic DNA (0.5 µg) was bisulfite converted and prepared for targeted deep sequencing. Four amplicons were designed to cover all CpG dinucleotides (numbered 57 to 95) between the 3′ end of the CpG island and the *Alu* element containing the GAA triplet-repeat in intron 1. The amplicons were dual-indexed and pooled to create a library which was sequenced using the Illumina MiniSeq platform. n = 1000 sequence reads were used to calculate the percentage of methylated cytosines at individual CpG dinucleotides (CpGs 57 to 95) and plotted with LOWESS smoothing to generate trendlines. FRDA-DMR methylation values were calculated using n = 1000 sequencing reads of a single amplicon containing CpGs 72 to 82. The methylation panels depicting FRDA-DMR methylation were generated by stacking the sequence reads (n = 300 rows), with columns representing the n = 11 CpG dinucleotides in the FRDA-DMR, and marking each coordinate black if methylated and white if unmethylated (the individual reads [rows] were sorted for high methylation at the bottom). Note that for ease of visualization FRDA-DMR methylation panels were generated with n = 300 reads, however, all data were analyzed at n = 1000 sequence read depth.

### CRISPR-mediated DNA methylation of the FRDA-DMR

An all-in-one plasmid system, pdCas9-DNMT3A-PuroR_v2, a gift from Vlatka Zoldoš (Addgene plasmid #74407) expressing a gRNA separately from a dCas9-DNMT3A fusion protein was used to epimodify the FRDA-DMR in HEK293T cells. gRNA target sequences were designed to target *FXN* intron 1 in the vicinity of FRDA-DMR using the web tool CHOPCHOP^62^. All Multiple gRNAs were synthesized by oligo annealing^63^, and cloned into pdCas9-DNMT3A-PuroR_v2 using Bbs I and tested for their ability to guide dCas9-DNMT3A to the FRDA-DMR. The gRNA overlapping CpGs 66 and 67 (depicted by an arrow alongside the X-axis in Fig. 5C; gRNAF: 5′-CACCGGGCACGGGCGAAGGCAGGGC-3′ and gRNAR: 5′-AAACGCCCTGCCTTCGCCCGTGCCC-3′) resulted in DNA methylation of the FRDA-DMR that was comparable to what is seen in FRDA and was therefore chosen. A “scramble” gRNA, with no target in the human genome, was used as a control (ScrF: 5′-CACCGCGAGGCGCTACGCGTGGACT-3′ and ScrR: 5′-AAACAGTCCACGCGTAGCGCCTCGC-3′). The transfection protocol previously reported^39^ was followed, with slight modifications. Briefly, HEK293T cells were seeded in 6-well plates and incubated at 37 C for 24 h. Cells were transfected at ~ 90% confluency with 1ug of plasmid DNA using Lipofectamine 3000 following the manufacturer’s protocol (Life Technologies). Three days post-transfection, cells were treated with 2.5 μg/ml puromycin. DMEM containing puromycin (2.5 μg/ml) was replenished every 24 h until 7 days post-transfection. Cells were then harvested, and DNA and RNA were extracted using the AllPrep DNA/RNA Mini Kit (Qiagen).

### Statistical analyses

Statistical tests were performed using GraphPad, Prism v9. Two-tailed, unpaired student’s *t* test, ordinary one-way ANOVA, or two-way ANOVA was used to compare means where appropriate. Linear correlations were evaluated with Pearson correlation coefficient where appropriate, and the Benjamini and Hochberg method^64^ of false discovery rate was used to account for multiple linear regression comparisons where appropriate. Specific statistical tests used, number of replicates, statistical values, including degrees of freedom and *p* values are included in the figure captions accompanying the data.

## Supplementary Information

Supplementary Information.
