# Supplementary material for: DNA methylation in Friedreich ataxia silences expression of frataxin isoform E
Source: Sci Rep. 2022 Mar 23;12:5031. doi: 10.1038/s41598-022-09002-5 (PMC8943190; doi:10.1038/s41598-022-09002-5)

Tables

Table S1. Patient demographics

| Characteristic | n=32 |
|---|---|
| Female, No. (%) | 17 (53%) |
| Age of onset, yr Median (range) | 9.5 (2-22) |
| GAA1, triplets Median (range) | 742 (53-1000) |
| GAA2, triplets Median (range) | 1000 (570-1367) |

Table S2. GAA repeat lengths for neural progenitors and proprioceptive neurons

| | Sample | GAA1 | GAA2 |
|---|---|---|---|
| FRDA1 | progenitor | 377 | 771 |
| | neuron | 341 | 771 |
| FRDA2 | progenitor | 598 | 740 |
| | neuron | 598 | 740 |
| FRDA3 | progenitor | 416 | 837 |
| | neuron | 416 | 837 |

**Figure S1**

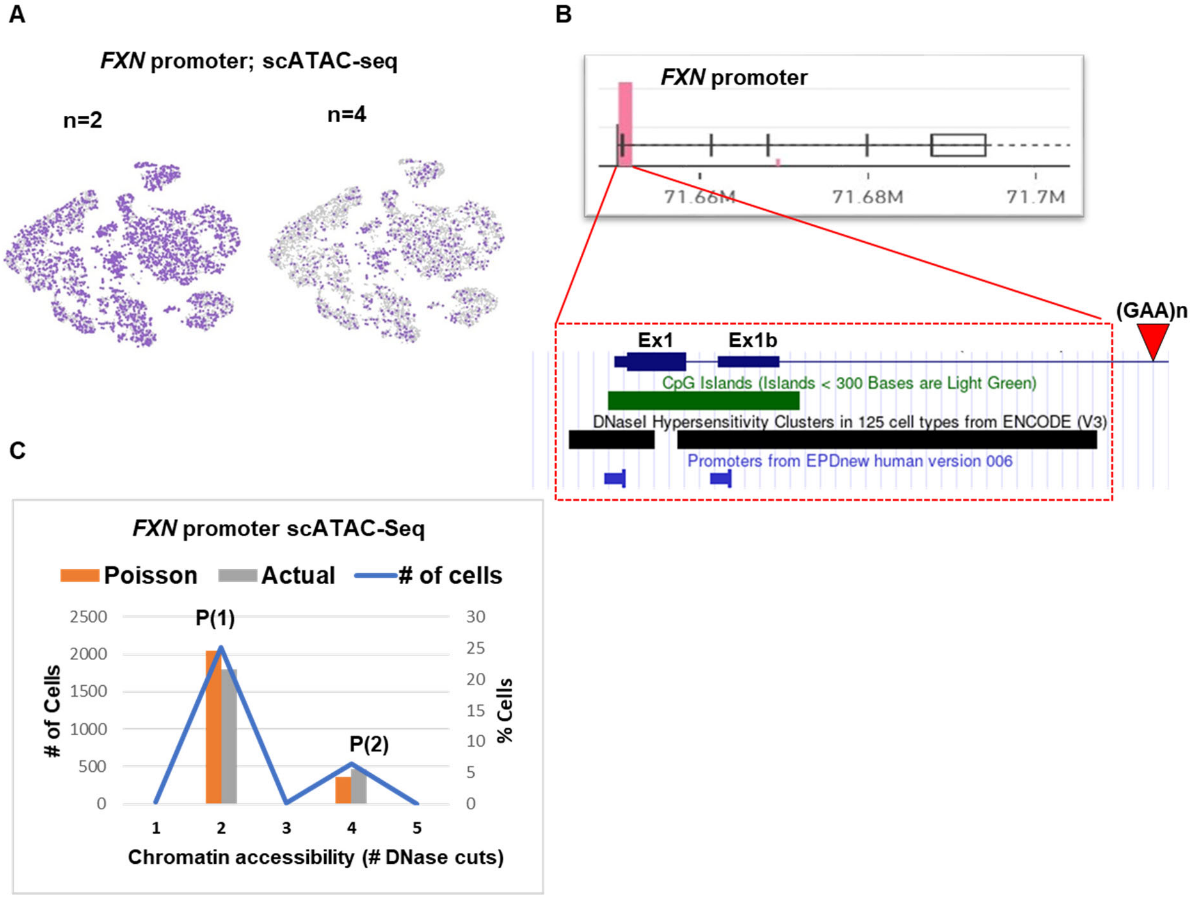


**Figure S1. The *FXN* promoter contains two discrete regions of open chromatin.** (**A**) Publicly available 10x Genomics scATAC-seq dataset of 10K PBMCs from a healthy donor. Cells with *FXN* promoter accessibility are displayed as purple spots overlaying 9,668 PBMC nuclei [gray background; t-SNE dimensional reduction]. (**B**) The *FXN* promoter is defined per default settings in Ensembl (hg19), which spans the canonical *FXN* promoter and extends into the 5' end of intron 1 (red box) thus encompassing the entire CpG island (green box), two DNase hypersensitivity clusters (black boxes), and both *FXN* promoters annotated by the EPDnew promoter database (blue bars). The relative locations of Exon 1 (Ex1), Exon 1b (Ex1b), and the GAA repeat (red triangle) are shown. (**C**) ~25% and ~5% of the cells show exactly 2 and 4 cuts respectively, with almost none showing 1 or 3 cuts [gray bars/right Y-axis, % cells (actual); blue line/left Y-axis, cell count (actual)] which, match the Poisson probabilities of 1 and 2 cuts, respectively [designated as P(1) & P(2); orange bars/right Y-axis, % cells (probability)] indicating that there are two distinct sites of chromatin accessibility, and that they are concordant and therefore in *cis*.

**Figure S2**

A

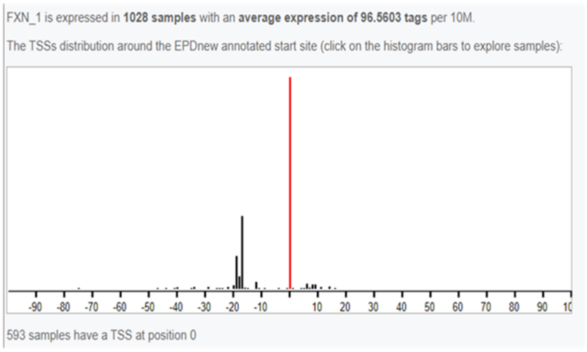


B

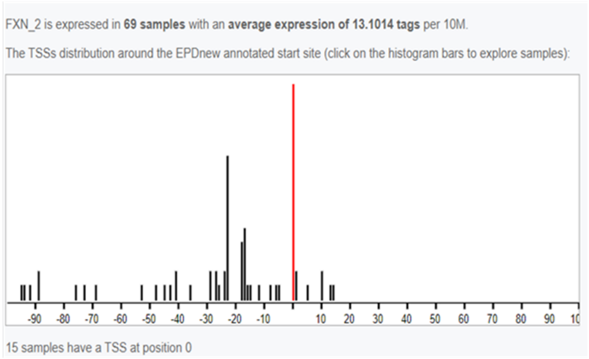


C

| | FXN_2 | |
|---|---|---|
| | **Sample** | **Expression (relative units)** |
| 1 | Astrocyte - cerebellum - donor2 | 30 |
| 2 | occipital pole - adult - pool1 | 26 |
| 3 | Astrocyte - cerebellum - donor1 | 20 |
| 4 | CD34+ Mobilized cell longPolyA rep1 | 16 |
| 5 | postcentral gyrus - adult - pool1 | 16 |
| 6 | Fibroblast - Choroid Plexus - donor1 | 13 |
| 7 | paracentral gyrus - adult - pool1 | 13 |
| 8 | medulla oblongata - adult - pool1 | 12 |
| 9 | IMR90 nucleus longPolyA rep2 | 11 |
| 10 | Pericytes - donor1 | 11 |
| 11 | SkMC cell longPolyA rep2 | 11 |
| 12 | HWP cell longPolyA rep1 | 10 |
| 13 | SkMC cell longPolyA rep1 | 10 |
| 14 | Small Airway Epithelial Cells - donor2 | 10 |
| 15 | testis - adult - pool1 | 10 |

**Figure S2. EPDnew promoter database (https://epd.epfl.ch//index.php) identifies 2 promoters at the *FXN* locus named FXN_1 and FXN_2.** (**A**) FXN_1 is the promoter for frataxin-M and is expressed in 1028 different cell types with an average expression of 96.5603 tags/10 million. The red line indicates position 0 which is -14bp relative to the A of the ATG start codon in exon 1 (position 0=71,650,685 per GRCh37/hg19). Of the total 1028 cell types, 593 have a TSS at position 0. (**B**) FXN_2 is the promoter for frataxin-E and is expressed in 69 different cell types with an average expression of 13.1014 tags/10 million. The red line indicates position 0 which is +279bp relative to the A of the ATG start codon in exon 1 (position 0=71,650,978 per GRCh37/hg19). (**C**) Of the total 69 cell types, 15 have a TSS at position 0.

**Figure S3**

**A**

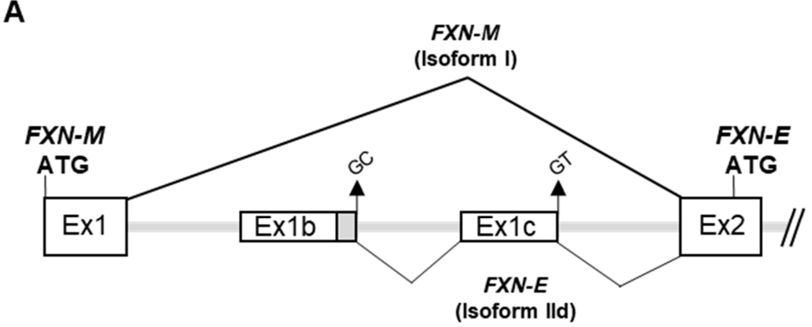


**B**

ggggtcgctccgggtacgcgcgctggactagctcaccccgctccttctcagggcggcccggcggaagcggccttgcaactcccttctctggttctc

ccggttgcatttacactggcttctgctttccgaaggaaaaggggacattttgtcctgcggtgcgactgcgggtcaag**gctgctggagtgcaatggt**

**gtaatcatagctcactgcagcctctacctctctggctcaagcaatcctcccacctcggcctcctgagtagctgggactacag**AGTTC

GAACCAACGTGGCCTCAACCAGATTTGGAATGTCAAAAAGCAGAGTGTCTATTTGATGAATTTGAG

GAAATCT...

**Figure S3.** ***FXN-E* isoform IId.** (**A**) A rare 4th isoform, IId, containing a second intronic exon between Ex1b and Ex2, Ex1c (corresponding with an annotated exon in GENCODE (71,658,377-71,658,477 per GRCh37/hg19), was observed in LBCLs. Ex1c spliced to the Ex1b variant containing the +18 nucleotides (gray box) with a non-canonical splice sequence GC and to Ex2 with the canonical splice sequence GT. (**B**) Sequence of the spliced exons Ex1b+18, Ex1c, and Ex2. Ex1b is displayed in lowercase text with the +18 nucleotides enclosed by a box. Ex1c is displayed in lowercase bold text and Ex2 is displayed in uppercase text. The “ATG” start codon in Ex2 is underlined.

**Figure S4**

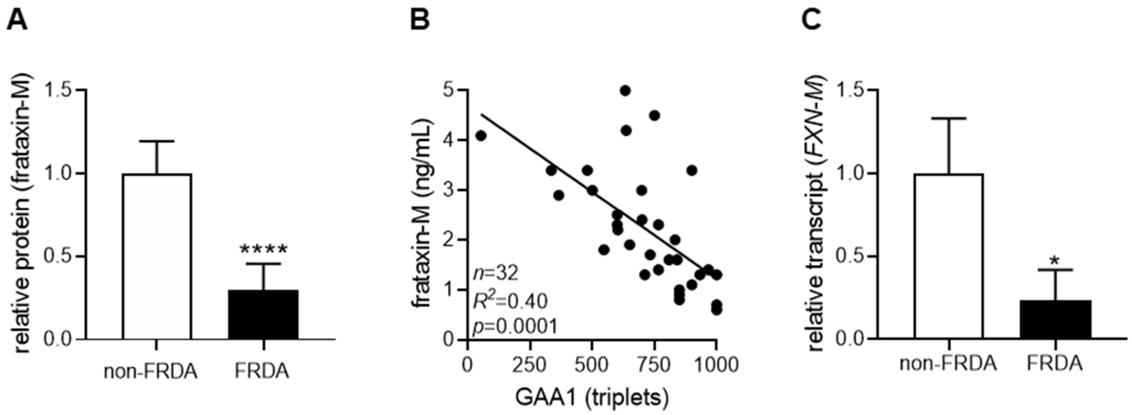


**Figure S4. Frataxin-M and *FXN-M* deficiency in FRDA.** (**A**) Frataxin-M protein in our cohort of FRDA and non-FRDA PBMCs (n=32 FRDA, n=11 non-FRDA; two-tailed, unpaired student's t-test, t=11.92, df=41, ****=$p$<0.0001). (**B**) Frataxin-M protein is inversely correlated with with GAA1 repeat length in our cohort of FRDA PBMCs (Pearson). (**C**) *FXN-M* transcript in non-FRDA and FRDA LBCLs (n=2 non-FRDA, n=4 FRDA; two-tailed, unpaired student's t-test, t=3.87, df=4, *=$p$<0.05)

**Figure S5**

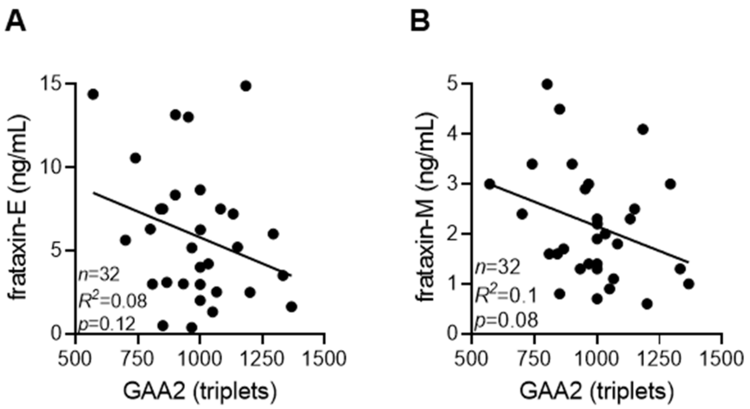


**Figure S5. Frataxin-E and frataxin-M deficiency does not correlate with the GAA2 allele in FRDA.** (**A**) Neither frataxin-E nor (**B**) frataxin-M protein correlates with GAA2 repeat length in our cohort (Pearson).

**Figure S6**

A

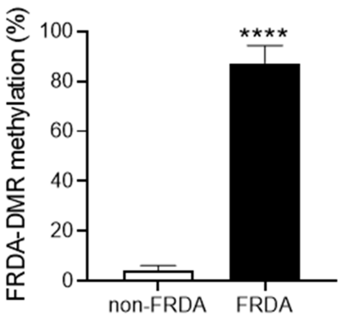


**Figure S6. Hypermethylation of the FRDA-DMR in FRDA PBMCs.** (**A**) FRDA-DMR methylation in our cohort of FRDA and non-FRDA PBMCs (n=32 FRDA, n=11 non-FRDA; two-tailed, unpaired student's t-test, t=42.69, df=44, ****=$p$<0.0001).

**Figure S7**

A

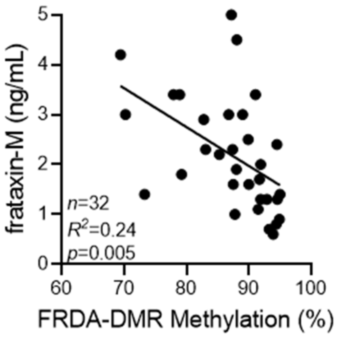


**Figure S7. Frataxin-M is inversely correlated with FRDA-DMR methylation in FRDA.** (**A**) Frataxin-M protein inversely correlates with FRDA-DMR methylation in our cohort of FRDA PBMCs (Pearson).

**Figure S8**

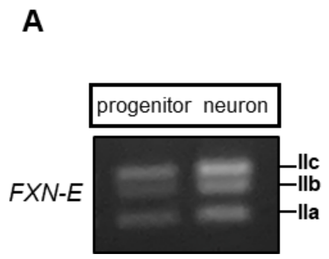


**Figure S8. Frataxin-E isoforms expressed in iPS-derived proprioceptive neurons and neuronal progenitors.** (**A**) Neuronal progenitors express *FXN-E* isoforms IIa, IIb,and IIc. Note: Uncropped gel image is included in Figure S14.

**Figure S9**

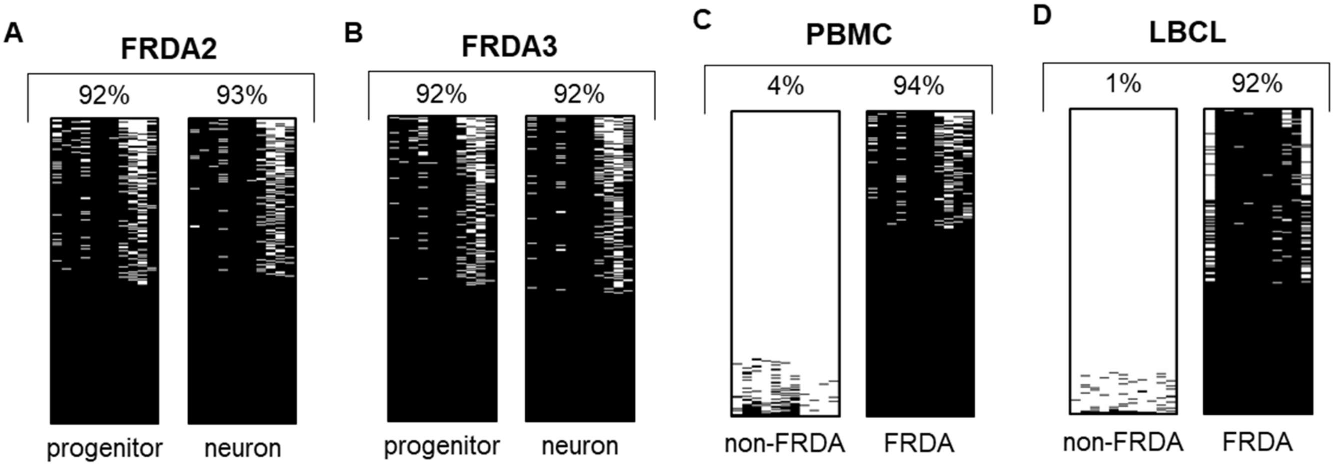


**Figure S9. Hypermethylation of the FRDA-DMR in FRDA iPS-derived proprioceptive neurons.** (**A**)(**B**) Hypermethylation of the FRDA-DMR seen in progenitors and neurons from the other n=2 patients not shown in Figure 3 is analogous to that seen in FRDA (**C**) PBMCs and (**D**) LBCLs.

**Figure S10**

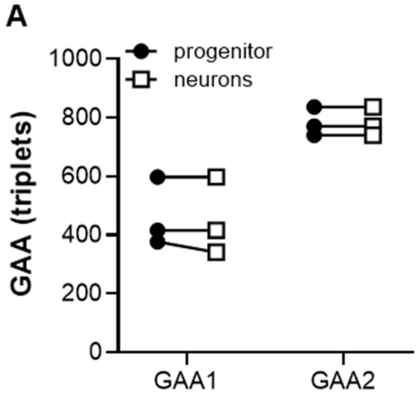


**Figure S10. Stability of the expanded GAA repeats through neuronal differentiation.** (**A**) GAA repeat lengths for all n=3 progenitors and neurons from FRDA patients.

**Figure S11**

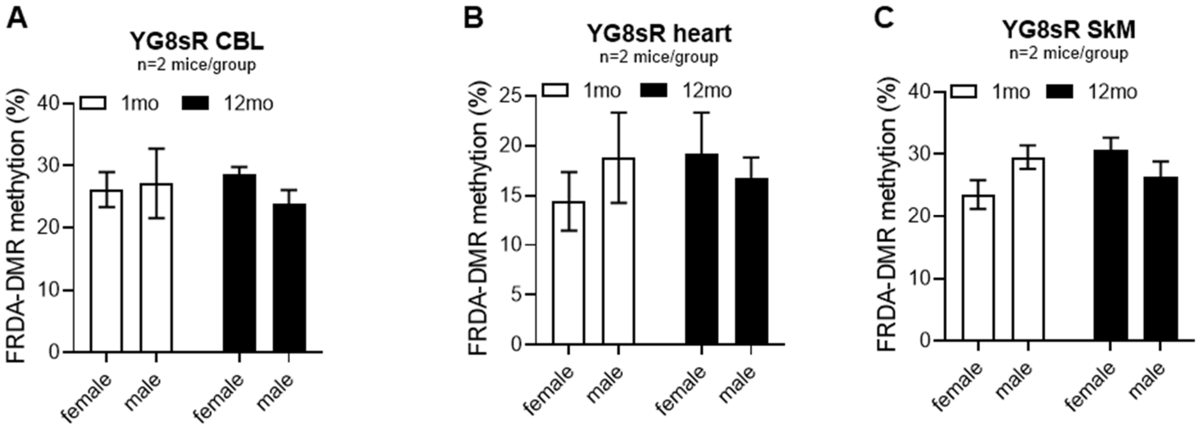


**Figure S11. FRDA-DMR methylation in FRDA is independent of age and sex.** (**A**)(**B**)(**C**) FRDA-DMR methylation in the YG8sR-480 mouse between sexes and across tissues. CBL=cerebellum, SkM=skeletal muscle (2way ANOVA, all comparisons are n.s.).

**Figure S12**

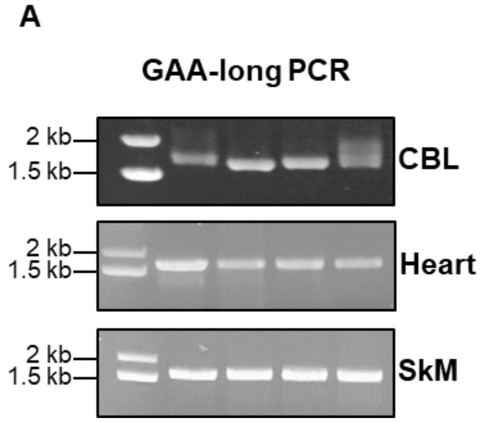


**Figure S12. Stability of the expanded GAA repeats in YG8sR mouse tissues.** (**A**) GAA long PCR products indicate the repeat length in n=4 representative YG8sR mice for CBL, heart, and SkM. Note: Uncropped gel images are included in Figure S14.

**Figure S13**

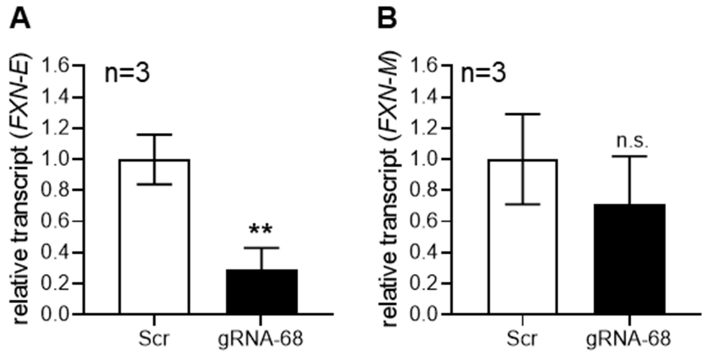


**Figure S13. DNA hypermethylation of the FRDA-DMR silences *FXN-E.*** (**A**) *FXN-E* and (**B**) *FXN-M* transcript levels measured with RT-qPCR (n=3 in all groups; two-tailed, unpaired student's t-test; (A) t=5.82, df=4, **=$p$<0.01. (B) t=1.23, df=4, n.s.=not significant).

# Figure S14

Fig. 1B

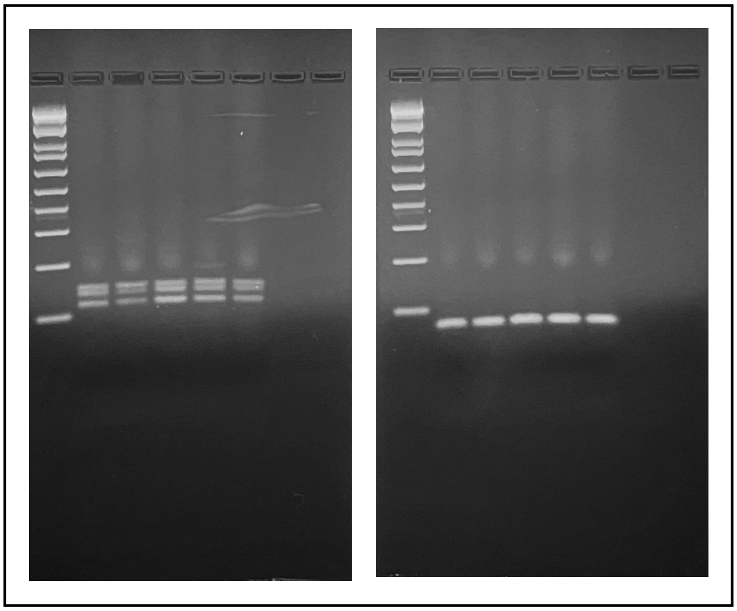

Fig. 3A

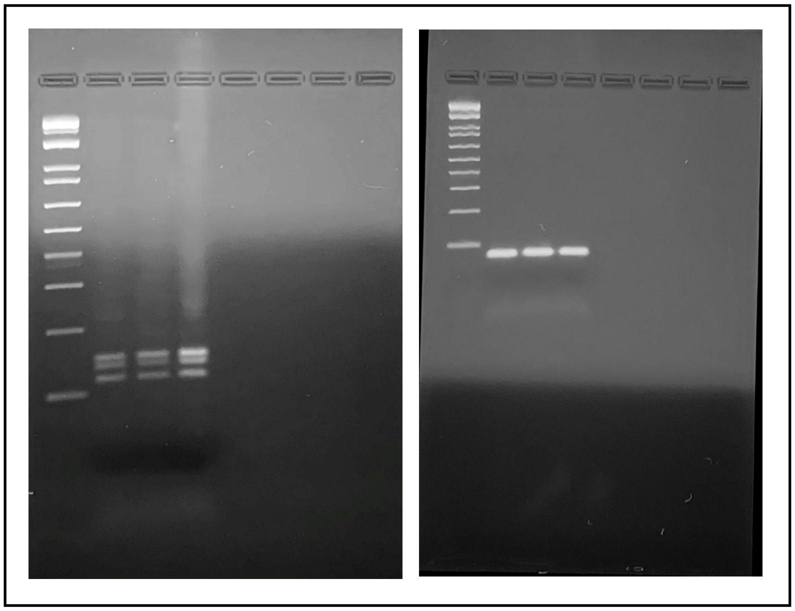

Fig. 4A

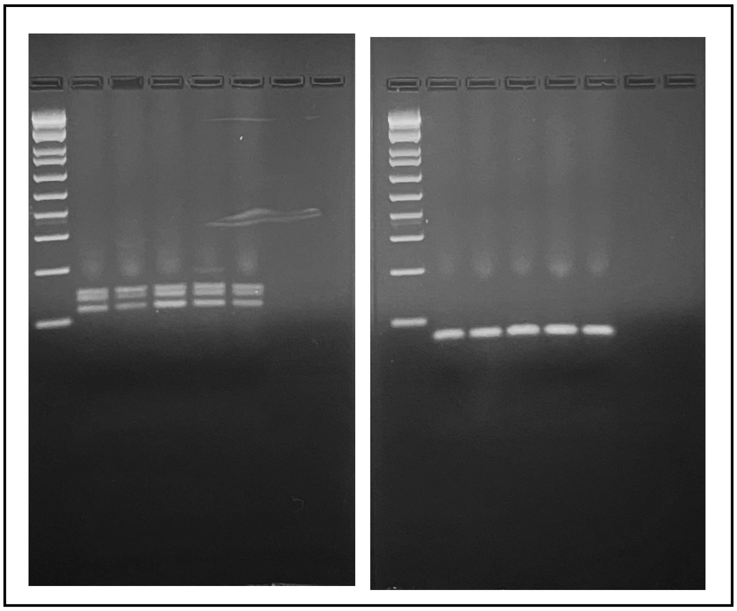

Fig. S8A

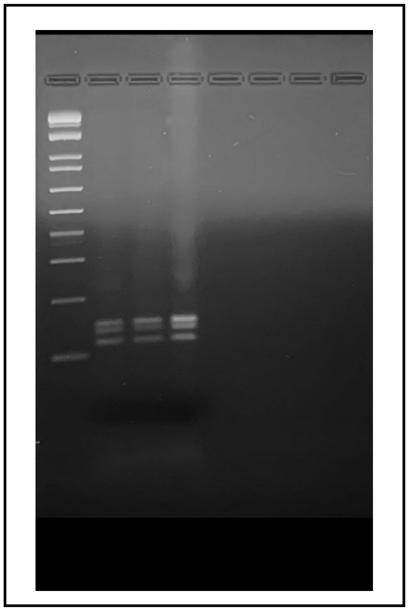

Fig. S12A

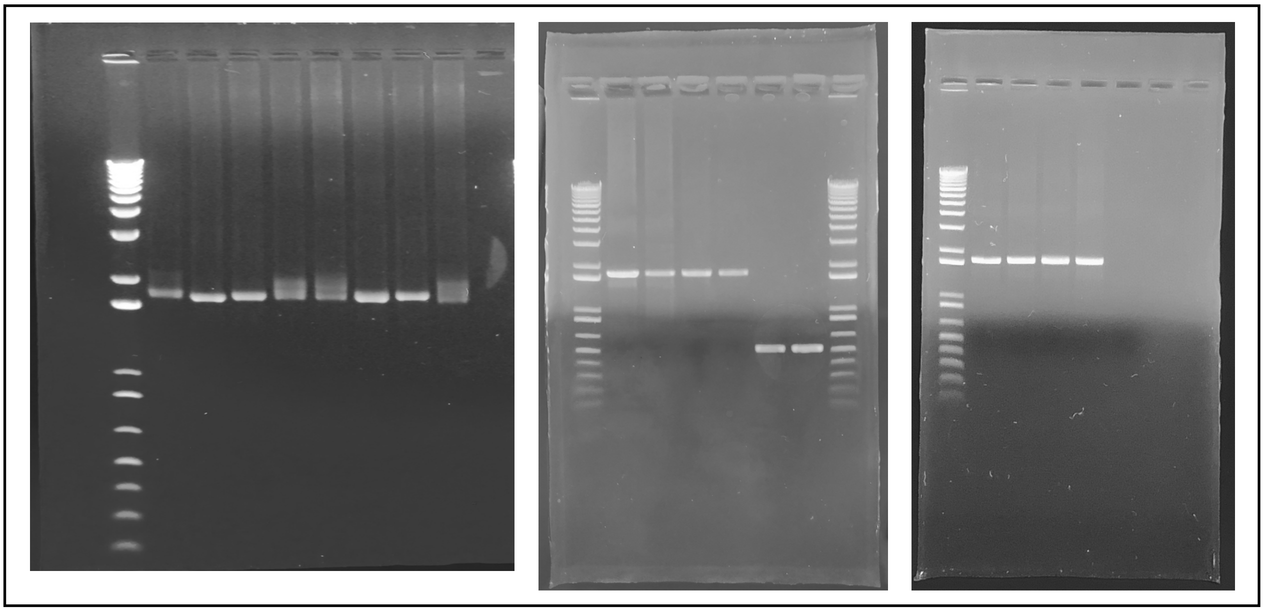

Supplement: Supplementary file 1 — Supplementary Information. [file 41598_2022_9002_MOESM1_ESM.pdf]
